# Forensic Analysis of Decapitation in an Agricultural Worker: A Case Report on Farm Tractor-Related Fatality

**DOI:** 10.7759/cureus.109711

**Published:** 2026-05-26

**Authors:** Ivana M Andric, Zivana Slovic, Milos Todorovic, Milena Vuletic, Ksenija Bosnjakovic, Vladimir Sebek, Katarina Vitosevic

**Affiliations:** 1 Department of Forensic Medicine, Faculty of Medical Sciences, University of Kragujevac, Kragujevac, SRB; 2 Department of Forensic Medicine and Toxicology, University Clinical Center, Kragujevac, SRB; 3 Department of Pathology, Faculty of Medical Sciences, University of Kragujevac, Kragujevac, SRB; 4 Department of Criminalistics, Faculty of Law, University of Kragujevac, Kragujevac, SRB

**Keywords:** agriculture, decapitation, farm tractor, forensic pathology, occupational hazard

## Abstract

The agricultural sector relies heavily on farm workers who operate tractors and other associated heavy machinery, mostly attached to the vehicle itself. With that in mind, they are at high risk of occupational and road traffic-related fatalities. Decapitation resulting from these accidents is an exceptionally rare outcome.

A 61-year-old male was found decapitated on a farm field he was working at, next to a running farm tractor. The body was delivered with a decapitated head, of which the spinal cord was striking through. Also, the left earlobe was completely amputated. On the upper part of the back, the print of the tractor tire pattern was seen, and on the right leg, a similar pattern was seen. Internal findings showed significant brain edema, bilateral subdural and subarachnoid hemorrhage, and complete horizontal laceration of the pontomedullary junction and basilar artery. The neck organs, along with the tongue, were mostly destroyed, with a partly destroyed pharynx, esophagus, and hard palate. Toxicological analysis detected an alcohol concentration of 3.03 mg/mL in blood, while other substances were excluded.

In each case of decapitation, it is necessary to find vital reactions and exclude postmortem mutilation. The cause of death was decapitation, and the manner of death was determined to be accidental. In this case, without witnesses, the autopsy was vital in the reconstruction of the crime scene based on the injuries involved. Macroscopic and microscopic examination of the wound edges showed that the decapitation occurred while the victim was alive, which is a crucial aspect of the autopsy process.

## Introduction

The agricultural sector ranks among the most hazardous industries worldwide. Machinery, tractors, heavy lifting, hand tools, farm animals, pesticides, and other chemicals predispose agricultural workers to injuries. Among them, farm tractors and other associated heavy machinery connected to precarious situations are a leading cause of serious and fatal injuries to farm workers [1,2]. Tractor-related agricultural injuries are usually severe and polytraumatic. Injuries in the agriculture sector represent an important burden of disease, often leading to permanent physical impairment, long-term disability, and death. The majority of farm workers are injured while operating tractors, which can cause trauma to the head, torso, spine, and extremities. These lesions can be extremely severe, often leading to amputation, either primarily or as a consequence of surgical treatment and extensive tissue damage. In many cases, survivors require prolonged hospitalization, multiple reconstructive procedures, and lengthy rehabilitation, which additionally contributes to the socioeconomic burden on rural communities and healthcare systems [3,4].

Also, older model tractors, which are usually used in Serbia, come with increased risk for injuries, as older model tractors lack safety equipment [3,4]. Many of these vehicles are not equipped with rollover protective structures (ROPS), seat belts, enclosed cabins, or modern braking and stabilization systems. In addition, inadequate maintenance and prolonged use of outdated machinery further increase the probability of mechanical malfunction and loss of vehicle control. The second problem is that, in the majority of cases, farm workers take on complex or hazardous tasks while working alone [5]. Working in isolated rural areas often delays emergency response and medical assistance, thereby worsening injury outcomes and increasing mortality rates.

Demographic factors such as age and psychosocial factors are also important, including greater fatigue, lapses in concentration, inexperience, carelessness, inaccuracy, distraction, consumption of alcohol, and reduced perception of danger. Elderly farmers are particularly vulnerable because of slower reflexes, impaired mobility, chronic illnesses, and prolonged exposure to physically demanding labor. Younger and less experienced workers, on the other hand, may underestimate occupational hazards and engage in unsafe practices during machinery operation. Furthermore, long working hours, seasonal pressure, and adverse weather conditions contribute significantly to occupational stress and reduced alertness [3,4].

Serbia's agricultural sector relies heavily on farm workers who operate tractors and other associated heavy machinery, mostly attached to the vehicle itself. With that in mind, they are at high risk of occupational and road traffic-related fatalities. Mechanization has certainly made farm work more manageable for agricultural employees by reducing heavy workloads in many demanding tasks; however, at the same time, high noise exposure, environmental pollution, vibrations, insufficient operator training, and the lack of adequate safety mechanisms can turn machines into a major source of danger within the farm environment. In rural regions of Serbia, tractors are frequently used not only in agricultural fields but also on public roads for the transportation of goods, crops, and equipment, which additionally increases the risk of collisions and fatal traffic accidents.

There are numerous occupational risks in the agricultural sector, such as the use of equipment and machinery requiring specific training, location in dangerous terrains due to slopes and gradients, and the multifunctionality associated with agriculture. Farm workers are often simultaneously exposed to mechanical, physical, chemical, and environmental hazards. Uneven terrain, poor visibility, muddy surfaces, and unstable loads significantly increase the likelihood of overturning and crushing incidents. The most frequent cause of injury is an accident involving a farm tractor, mainly caused by tractor rollover, followed by falling from the tractor and being run over by the tractor, while traffic accidents are less common [4]. Tractor rollovers are particularly associated with fatal outcomes because of the enormous weight of the vehicle and the absence of protective structures in older tractor models. Such accidents frequently result in multiple traumatic injuries involving the skull, cervical spine, thorax, abdomen, and limbs.

Decapitation [6] is defined as an amputation of the head, which leads to loss of brain function due to ischemia. Depending on the mechanism of injury, decapitation may be complete or incomplete and is generally associated with catastrophic disruption of the cervical spine, spinal cord, vascular structures, and surrounding soft tissues. Although nowadays it is mainly used as a means of euthanasia in experimental work with rodents, because it does not chemically contaminate tissues or alter tissue architecture, cases of decapitation are still sporadically present in forensic medicine. Such injuries are most commonly associated with high-energy trauma, including railway accidents, industrial injuries, motor vehicle collisions, and, rarely, agricultural incidents. Accidental decapitations in agricultural settings are exceptionally uncommon. Due to the rarity and severity of these injuries, they are of particular forensic and medico-legal importance, especially in determining the mechanism and circumstances of death. In this article, we report on an unusual case of a farmer who was decapitated while working alone in a farm field.

## Case presentation

Illustration of the scene

**Figure 1:**
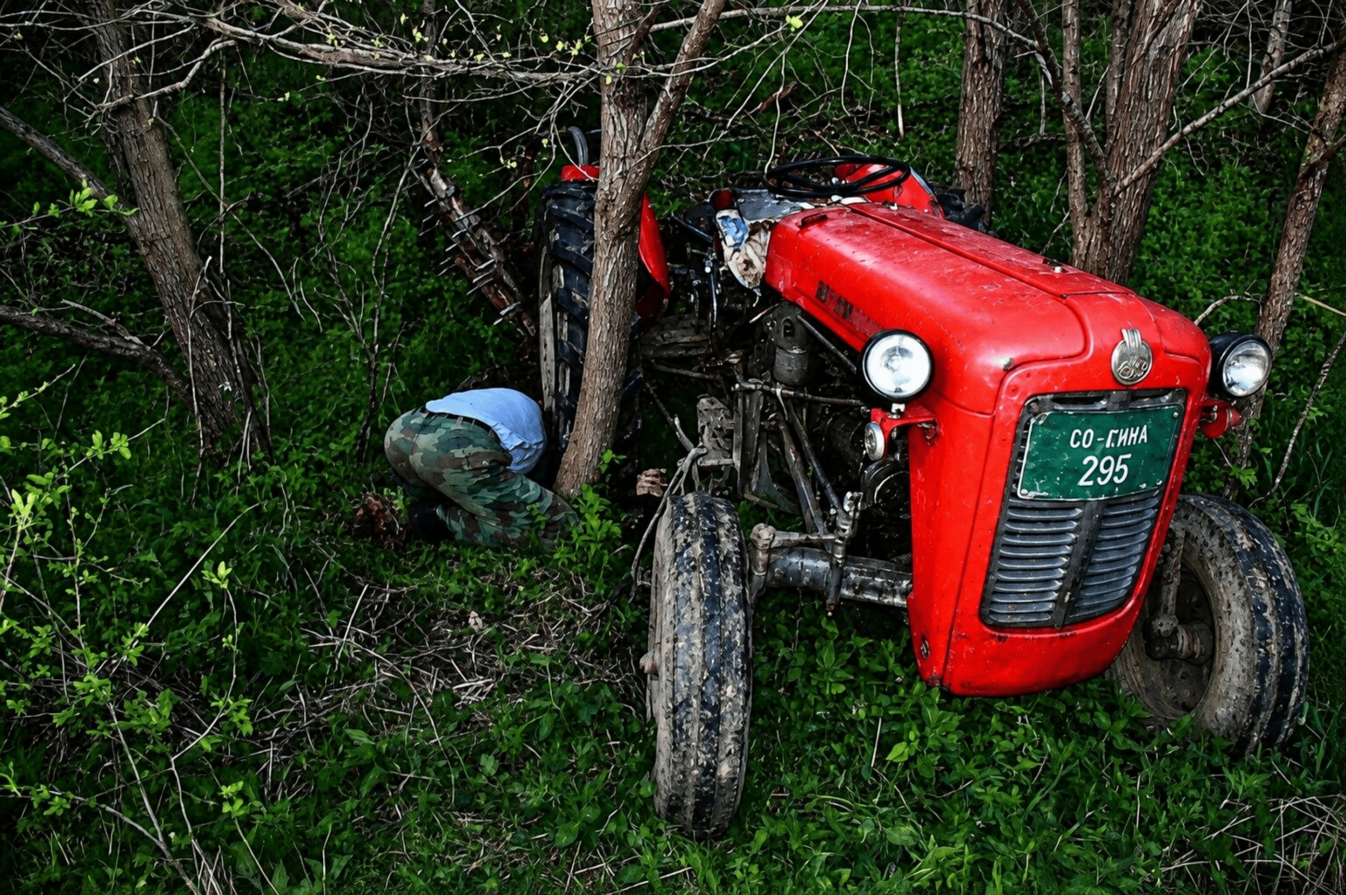
Location of the event scene

The victim was a 61-year-old male farmer who had been working during the entire Saturday, without any other witnesses. He was discovered at dusk, in a kneeling position facing downwards, in a farm field between the farm tractor and a tree trunk, with his body next to the bottom right tractor tire (Figure 1) and a tree trunk. The tractor was still on, as the motor was working when the police came to the event scene. His completely decapitated head was found next to the right arm of the body, in front of the rear right tractor tire. No significant amount of blood was found at the scene, which was unusual for decapitation. Approximately 5 meters away from the tractor, an overturned handmade trailer was observed.

Autopsy report

**Figure 2:**
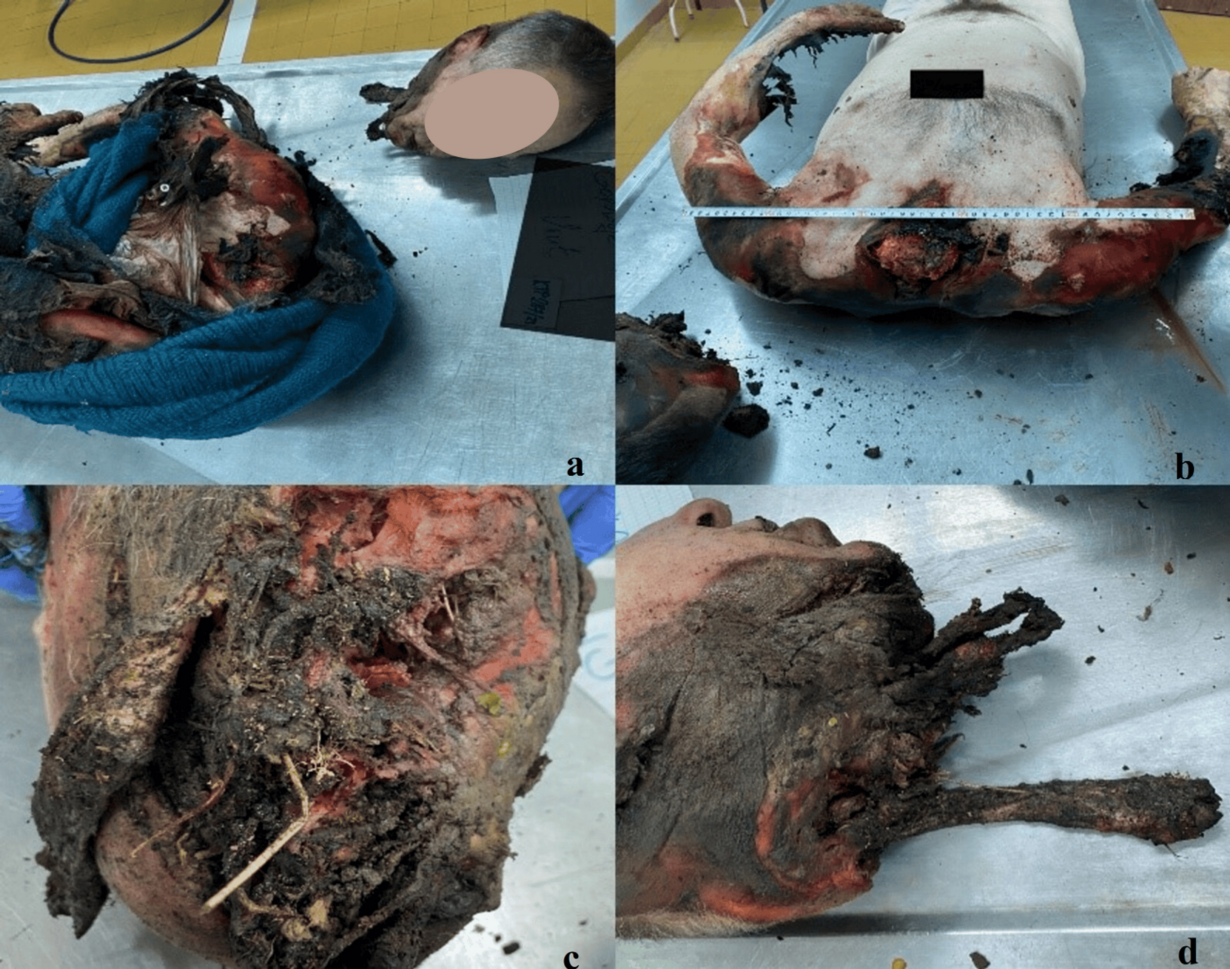
(a) Decapitated body; (b) Wound appearance on the body; (c) Wound appearance on the back of the head; and (d) Spinal cord protrusion.

The body, with a length of 164 cm and a head of 26 cm (in total 180 cm), was sent to an autopsy, completely decapitated at the level of the third cervical vertebra. The margins of the head and neck wound were irregular and ragged, with tears and pull force injuries stained with dirt and grass particles (Figure 2). Over a large area, including both upper arms, shoulders, and the proximal part of the back, the skin was diffusely altered, dark brown and dark red in color. The body was dressed in a blue sweater and a grayish-white button-down shirt, both of which were bedraggled with dirt and splashes of diesel oil, with massive tears, scowls, and rips on the top and mostly right side of the clothes. The bottom was dressed in an old army uniform and traditional rubber shoes that locals in this region of Serbia commonly used for working with hay straws and mud, corresponding to the field where the farmer was found.

**Figure 3:**
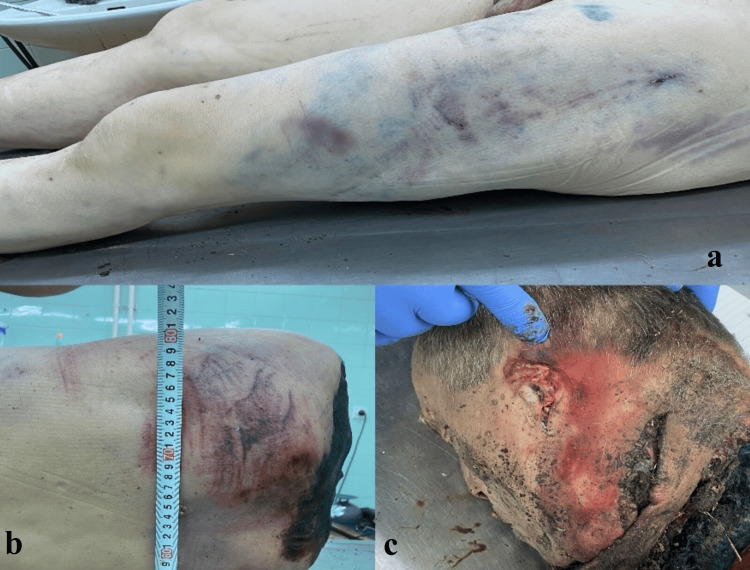
(a) Imprint of the tire on the left thigh; (b) Imprint of the tire on the back; and (c) Left ear lobe avulsion.

Based on our examination of the head wound, we observed that 23 cm of the stripped spinal cord protruded from the decapitated head, with the direction of the wound extending from front to back at an acute angle. On the front part of the left thigh, the imprint of the tire was found, along with the decollement in the internal findings (Figure 3a). On the upper back, on the outskirt of the neck, the imprint of the tractor tire pattern was observed (Figure 3b). The left earlobe was also completely amputated (Figure 3c), but no remains of the earlobe were found at the crime scene.

**Figure 4:**
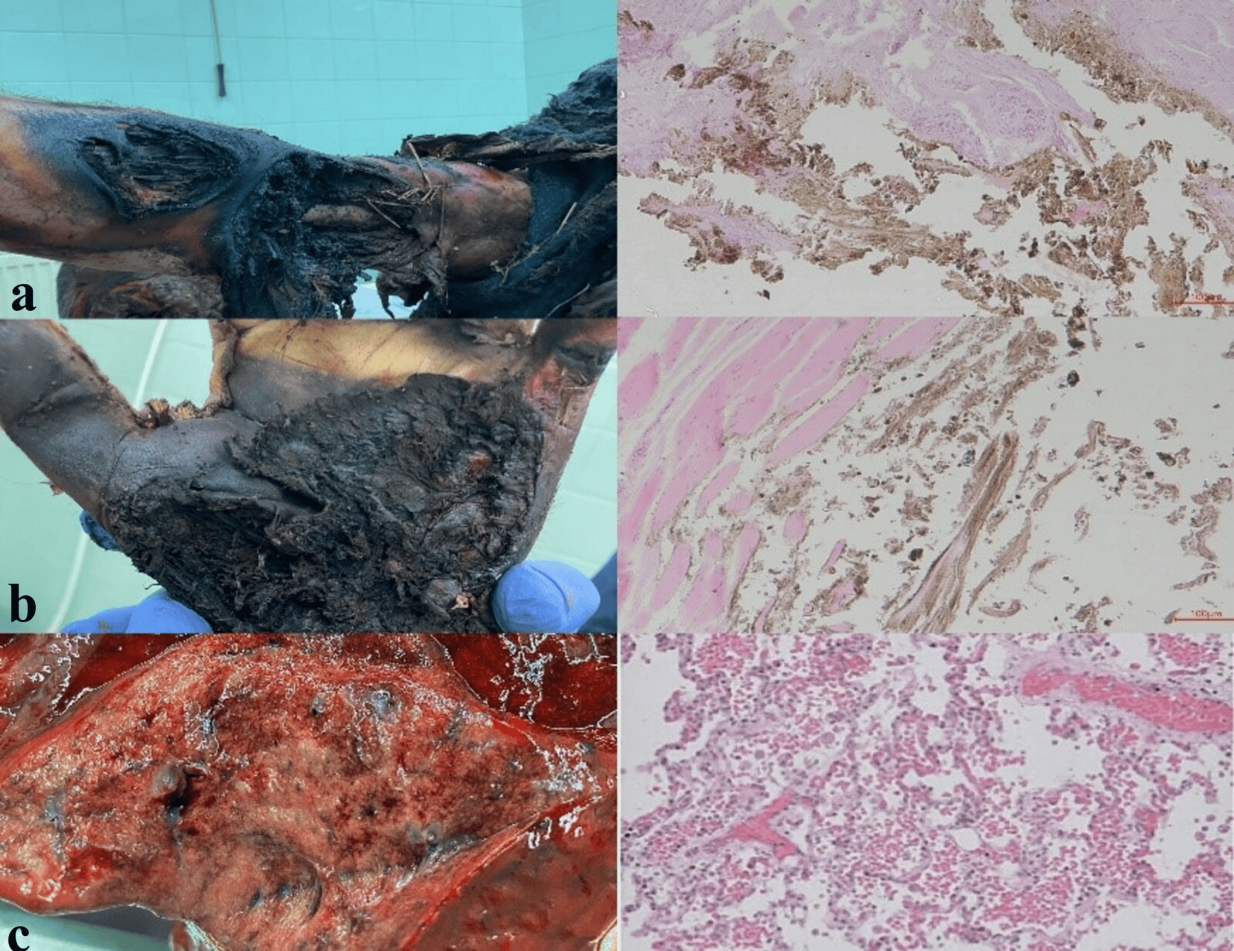
(a,b) Friction burns and histological findings of the area and (c) Blood aspiration in the lungs and histological findings of the area.

Within the stiffened fists due to rigor mortis were grass and hay strands. There was also significant tissue destruction on the inner surfaces of the right upper and forearm and the left forearm, with shredded, black-stained parts of the tissue that, upon washing and further examination, resembled friction burns, due to the contact with the moving rear tire. However, the staining of the tissues raised the question whether hot diesel contributed to the injuries on the arms, making them scalds. Histological examination of the sampled tissues showed nonspecific findings, including a minimal presence of inflammatory cells (Figures 4a, 4b), without formation of a significant inflammatory infiltrate. Hemorrhage was absent or only minimally present within the examined tissue sections, with no relevant extravasation into the surrounding structures. Overall, the microscopic findings did not demonstrate features of a pronounced vital tissue reaction and are consistent with an acute traumatic event occurring in a very short perimortem interval.

Internal examination revealed significant brain edema (brain weight: 1615 grams), bilateral subdural and subarachnoid hemorrhage, and complete pontomedullary and basilar artery horizontal laceration. The neck organs, along with the tongue, were mostly destroyed, including parts of the tongue, pharynx, esophagus, and hard palate. The larynx, hyoid bone, thyroid cartilage, and thyroid gland were not delivered with the body. The other findings showed blood aspiration in the lungs (Figure 4c); the pneumothorax probe was determined to be negative, but the air embolism test was positive and measured an amount of 18 ml of air by the aspirometer. Additionally, multiple fractures of the left ribs and traumatic decollement on the front side of the left thigh were found.

Toxicological analysis detected an ethyl alcohol concentration of 3.03 mg/mL in the blood using a standardized gas chromatography method. No other substances were detected. The cause of death was determined to be decapitation, and the manner of death was ruled accidental.

## Discussion

Decapitation is a rare finding in forensic practice and is most commonly associated with high-energy trauma. In addition to the historical usage of the guillotine for decapitation, primarily in cases of homicide, which have often been depicted in art and paintings [6] throughout history, homicides involving decapitation are usually connected with pathological jealousy or mob activities and are most commonly performed postmortem to dispose of the body [7].

Suicidal decapitation cases are mostly associated with train traffic railways [8] or instances of hanging from a great height [9], characterized by the drop of the body. There have been rare instances of accidental decapitation in traffic-related accidents [10-12], primarily involving railway traffic, but also a case of an air traffic accident [13] was described in the literature. Another factor contributing to our conclusion that the manner of death is accidental is the head wound appearance, which is compliment to the findings of Tsokos et al. [14]. Similar to cases of train decapitation, the wound edges caused by the tractor tire were irregular and ragged, with tears and pull force injuries stained with dirt and grass particles. The wound edges were not sharp and straight but angled, as described by Byard and Gilbert [15], and in contrast to the other decapitation wounds sustained with heavy machinery, hanging, and ligatures, where clean and clear wound edges were noted in cases involving log splitters and train tracks [16,17].

In our case, with no witnesses at the scene, it was crucial to reconstruct the incident by examining the injuries and determining their vitality. By analyzing the injuries contained, it was necessary to determine the mechanism and sequence of injuries and decapitation, thereby concluding the manner of death, as there were no witnesses and no signs of altercation. The initial hypothesis proposed that the farmer fell out of the seat or that a turnaround of the tractor led to the decapitation. This hypothesis was excluded based on the injuries and the crime scene layout. Based on the wounds observed on the body, it appears that the tractor was moving backwards, striking the victim's left thigh with the rear tire. As a result, the victim rotated and fell backward, and the rear tire passed over the back of the body. The morphology of the neck wound, characterized by irregular, ragged edges and contamination with environmental material, is consistent with high-energy blunt force and traction, rather than sharp force injury. This supports an accidental mechanism involving heavy machinery. Steep and uneven terrain, characteristic of mountainous and hilly rural regions, represents one of the most significant risk factors for tractor overturn incidents. Agricultural tractors operating on slopes are exposed to altered centers of gravity, reduced stability, and increased susceptibility to lateral and rear overturns, particularly during turning maneuvers, transportation of heavy loads, or operation on wet and unstable ground surfaces [4]. Numerous epidemiological studies [1-5] have identified tractor rollovers as one of the leading causes of fatal occupational injuries in agriculture, with the risk being markedly higher in areas where agricultural activities are performed on steep inclines and poorly maintained field roads.

From a biomechanical and forensic engineering perspective, the fatal injury mechanism is consistent with a high-mass agricultural tractor run-over event involving substantial compressive, traction, and shear forces. The vehicle involved was an IMT Ferguson 533, characterized by a diesel 3-cylinder engine producing approximately 35 hp, an operating weight of approximately 1,500 kg, a rear-wheel-drive configuration, and a wheelbase of approximately 183 cm, representing the distance between the front and rear axle centers. The tractor length is approximately 296 cm, with relatively narrow front wheels ( tire width 12.7 cm, rim diameter 40.6 cm ) and substantially larger rear agricultural traction tires commonly measuring (tire width 28.4 cm, rim diameter 71.1 cm). The overall external tire diameter (including the rubber tire, not just the rim) is 120 cm. The large rear wheel diameter, deep tread profile, and high torque transmission significantly increase the potential for concentrated compressive and tangential forces during wheel-body contact, particularly on unstable agricultural terrain. Manufacturer specifications indicate movement speeds ranging from approximately 2-22 km/h, depending on gear selection and engine rotation speed, with low-speed gears capable of generating substantial traction force and prolonged wheel-ground contact. In the present case, the accident occurred in a rural agricultural environment with uneven terrain conditions, factors recognized as important contributors to tractor instability and altered center-of-gravity dynamics. Agricultural tractors possess a relatively elevated center of gravity and limited lateral stability, especially during turning maneuvers, operation on inclined surfaces, or low-speed movement under high traction loads. Under such circumstances, loss of operator balance or a fall beneath the rear wheel may result in prolonged wheel-body interaction rather than isolated blunt impact trauma. The substantial mass of the rear wheel assembly, combined with rotational traction forces and vehicle weight, likely generated extreme cervical compression associated with tangential traction and rotational shear stress, producing catastrophic craniocervical dissociation and complete decapitation. The extensive soft tissue destruction and cervical skeletal disruption observed during autopsy support a combined crush-avulsion biomechanical mechanism rather than a sharp-force injury pattern. The scene investigation, vehicle characteristics, injury distribution, and autopsy findings strongly support a fatal tractor-associated rear-wheel run-over mechanism.

The severity and fatal outcome of such accidents are strongly associated with the absence of protective safety structures, including ROPS, safety cabins, and seat belts [4,5]. Tractors lacking these protective mechanisms provide minimal protection to the operator during overturn events, frequently resulting in crushing injuries, traumatic asphyxia, severe polytrauma, or death. Older tractor models, which are still widely used in rural and economically underdeveloped regions, often do not possess adequate safety equipment or are improperly maintained, further increasing the likelihood of fatal injuries [4].

An additional important contributing factor, also present in the current case, could be the solitary nature of agricultural work in rural areas. Farmers frequently operate machinery alone and in remote areas, without immediate supervision or assistance. Consequently, in the event of an accident, there may be a substantial delay in discovery, emergency response, and initiation of medical treatment [1]. Small-scale and family-operated farms are often exempt from occupational safety regulations due to operating in isolated environments with limited oversight. Farmers frequently work alone and are responsible not only for agricultural tasks but also for identifying risks and implementing safety measures without professional support. Economic pressures commonly lead to long working hours, increased risk-taking, and neglect of preventive practices such as regular machinery maintenance and use of protective equipment. Delayed rescue and prolonged entrapment beneath overturned machinery significantly worsen survival outcomes and increase the probability of fatal complications. Furthermore, the absence of witnesses may complicate the reconstruction of the accident mechanism and the precise determination of the chronology of injuries.

Advanced age, physical fatigue, prolonged working hours, inadequate training in safe tractor operation, and adverse environmental conditions additionally contribute to the increased incidence of tractor-related injuries in rural agricultural populations.

The injuries on both arms, sustained from holding onto the rear tire, were identified as burn marks due to friction and shearing forces. Microscopic examination of the wound edges indicated that the injuries were inflicted perimortem. The local vital reactions require more time for their manifestation, e.g., migration of polymorphonuclear leucocytes [18]. The main challenge in reconstructing the mechanism of injury is the localization of burn marks on the inner portions of the arms, as this finding complicates the interpretation of the injuries and their context, but it also contributes to the mechanism interpretation accuracy.

Clothes could have an additional impact on farm-related decapitation, as it was described in the case of Dermici et al., where the scarf was the main factor in the decapitation of a farm worker [19], but in our case, the clothes and the rubber shoes, although they did not directly impact the decapitation, could lead to slower reaction time due to lower agility.

**Figure 5:**
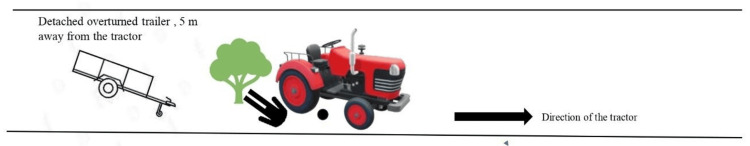
Reconstruction of the crime scene. The figure was created using PowerPoint (Microsoft Corporation, Redmond, WA, USA).

Based on the analysis of the injuries sustained, we have reconstructed the mechanism of injury, as depicted in Figure 5. The victim was operating the tractor in reverse in order to reattach the trailer that had overturned during the attachment. Upon dismounting the tractor, the victim failed to turn off the engine and engage the handbrake, resulting in the tractor continuing to move forward. The front tractor tire struck the front side of the victim’s left thigh, causing the inebriated victim to fall. Consequently, the rear tractor tire traversed over the back of the victim's body, as the presence of tire tread imprints on the back indicates that the body was run over, during which the head was decapitated, and the body stayed in a kneeling position. The arms exhibited injuries consistent with tearing and shearing forces, and burn marks due to friction, all contained likely due to the victim's attempt to hold onto the rear tire.

Alcohol is a major contributing accidentogenic factor, related to fatality [20] as it impairs motor skills, coordination, reflexes, and the time for reaction, vision, and consciousness. The sedative effects of alcohol can cause inattention, further reducing a person's ability to react to sudden changes in their environment, thereby increasing accident risk, especially when it comes to an appropriate response in potentially hazardous situations. The detected blood alcohol concentration of 3.03 mg/mL represents a markedly elevated ethanol level, exceeding standard legal intoxication thresholds in most jurisdictions and generally associated with severe psychomotor and cognitive impairment. The degree of impairment manifestation may vary according to individual tolerance and chronic alcohol consumption history, which could not be reliably assessed in the present case.

Regarding trace analysis, the lack of a significant amount of blood at the scene was explained by the porous nature of the terrain, such as soil, which easily absorbed most of the blood quickly. On non-porous surfaces like concrete or tile, blood tends to pool and remain on the surface, making it easier to collect and analyze. Different types of terrain can significantly affect the analysis of blood traces at a crime scene, influencing the accuracy and reliability of forensic conclusions. Understanding the interaction between blood and various terrains, particularly porous ones, is essential for accurate forensic interpretation and ensuring that critical evidence is not overlooked or misinterpreted.

**Figure 6:**
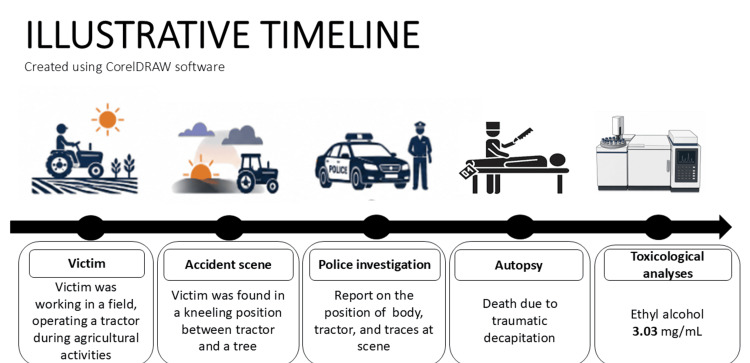
Illustrative timeline of events The figure was created using CorelDRAW (Corel Corporation, Ottawa, ON, Canada).

An illustrative timeline is presented in Figure 6.

## Conclusions

Agricultural decapitation cases are quite scarce. In each case of decapitation, it is necessary to find vital reactions and exclude postmortal mutilation. Even if there are not many blood traces on the scene, based on the forensic autopsy, we were able to determine the probable mechanism of injury based on the injuries sustained. In this case, without witnesses, the autopsy was key in the reconstruction of how the event unfolded, based on the injuries involved. Further studies should be involved about the force strength and other factors contributing to decapitation.
